# Modeling the Effects of Gap Cuts on Stand-Level Resilience in Financial and Carbon Sequestration Contexts

**DOI:** 10.1007/s00267-026-02418-z

**Published:** 2026-03-18

**Authors:** Jonathan Fibich, Alexander Lange, Thomas Clemen, Dominik Thom, Thomas Knoke

**Affiliations:** 1 Institute of Forest Management, Technical University of Munich, Freising, Bavaria Germany; 2 Department of Computer Science, Hamburg University of Applied Sciences, Hamburg, Germany; 3 Chair of Silviculture, Dresden University of Technology, Tharandt, Saxonia Germany

**Keywords:** Resilience, Quantification, Gap-cuts, Forest economics, Carbon sequestration

## Abstract

Climate change is amplifying disturbances in Central European forests, making resilience a key concept in forest management planning. Yet, the impacts and trade-offs of practices regarded as resilience-enhancing, such as pre-rotation age establishment of young trees in canopy gaps remain insufficiently quantified. Here, we adapt a recent quantification framework, using the post-disturbance recovery time of the present value of a given ecosystem service (*ES*) as stand-level resilience metric. Our novel, Monte-Carlo Simulation-based (MCS) approach models time series for two *ES* (net revenues and aboveground carbon sequestration) to quantify the impacts of disturbances under different gap-cut regimes, which we compare against a clear-cut baseline. Gap-cuts can notably enhance resilience, reducing average recovery times by up to 6.6 years (-15.7%) in the financial and 3.3 years (-21.3%) in the carbon sequestration context. Concerning the financial aspects, some gap-cut regimes small in gap size and late in timing could enhance resilience without associated losses in soil expectation value. However, most regimes, although dominating in the resilience metric, were associated with opportunity costs. These costs were less pronounced for carbon sequestration. We conclude that gap-cut induced regeneration can enhance resilience in both financial and carbon contexts, but their optimal timing and size depends on the prioritized ecosystem service. As the complexity of forest stand representations is inherently limited by our MCS-approach, further research should refine forest-value estimation through neural networks and mechanistic models, enabling richer representations of growth and disturbance dynamics and more detailed management regimes.

## Introduction

Forest ecosystems in central Europe are experiencing disturbances at an increasing rate (Seidl et al. 2011; Senf et al. 2018) and associated costs are expected to rise (Mohr et al. 2025). Large-scale storms, droughts and insect infections (see for example Möhring et al. 2021; Senf and Seidl, 2021) raise doubts concerning established forest-management strategies (Patacca et al. 2023), leading to changes and adaptations in species recommendations and desired forest structures (Bolte et al. 2009). Stakeholders and policy actors increasingly call for ‘resilience’ to be central to forest planning, and recent contributions aim to structure this fuzzy, multifaceted concept in the forest-management context (Lloret et al. 2024; Nikinmaa et al. 2020).

However, a standardized metric applicable for evaluating individual silvicultural adaptation measures is still lacking, which makes it difficult to compare and quantify their resilience-related effects (Linkov et al. 2014). To overcome this limitation, we present a new procedure that enables the quantitative assessment of resilience across management strategies. In particular, we evaluate gains and trade-offs arising from resilience-increasing silvicultural operations from a stand-level perspective.

Our approach builds on the concept of ‘engineering resilience’ (see Nikinmaa et al. 2020; Pimm 1984), focusing on the post-disturbance recovery of forest stands. Integrating future provision expectations, we utilize the present value of the assessed ecosystem service (*V*_*ES*_, see Section “The present value rationale”) as system variable (Lloret et al. 2024). Following Knoke et al. (2023), we use its post-disturbance recovery time as central resilience indicator: With *V*_*ES*_ representing the discounted expectation of future ecosystem-service flows, we focus our resilience assessment on *how quickly* the stand system regains its *capacity to generate provisions*. While this type of assessment has previously been limited to financial aspects (Knoke et al. 2023), we extend the same logic to aboveground carbon sequestration.

In central Europe, where major disturbance events are driven by storms and insects (Hanewinkel et al. 2008; Patacca et al. 2023), the establishment of regeneration cohorts within even-aged stands is seen as resilience-enhancing and is, in fact, oftentimes already utilized (Stokes et al. 2021). In most situations, this entails a stand density reduction to improve light conditions, which may involve partial harvests of stands in advance of their usual rotation age. The concept builds on the assumption that at least some portions of the regeneration cohorts will survive disturbances and, thus, will speed up the post-disturbance recovery process.

However, it is apparent that this practice might be associated with costs: If the establishment of regeneration cohorts involves partial harvests ahead of the usual rotation age, the overall performance with respect to a given ecosystem service (ES) might decrease compared to an even-aged clear-cut regime. The deviation from the original rotation age, which we assume to be more or less optimized to fulfill specific ES demands, may speed up the recovery process after a catastrophic event - but research on pre-rotation harvests (see for example Plotkowski et al. 2016) indicates there may also be a premium to be paid in form of a decrease in overall ES provision, similar to the premium paid to an insurance company.

Apart from this aspect of pre-rotation harvests, more mechanisms come to play in gap-cut stands that are likely to interfere with their resilience- and performance-related properties: Changes in the competition among single trees change growth dynamics, as the light- and nutrient-availability for the remaining stand suddenly increases (Lehtonen et al. 2023), whereas the regeneration cohort within the gap is dominated by the remaining older trees (Pacala et al. 1994). Furthermore, disturbance dynamics change as well, e.g., by remaining old trees lowering the regeneration cohorts’ height growth and thus the age-related wind-throw vulnerability (Schmidt et al. 2010). Finally, the option to receive natural regeneration within gaps can lower or even diminish the associated planting costs, increasing the overall economic performance. Facing those diverse and interrelated mechanisms, the combined effect of regeneration inducing gap-cuts on stand level resilience is non-trivial. We therefore aim to:

- quantify the effect of pre-rotation age, regeneration-inducing gap cuts on stand-level resilience, regarding both financial returns and carbon sequestration, and
- examine whether or not a stand manager has to pay a premium in ES provision in order to achieve those effects.

We acknowledge that resilience-related properties of gap-cuts have been studied before, focusing on diverse aspects such as forest structure (Kern et al. 2013), under-story vegetation (Tinya et al. 2025) or biodiversity (Laarmann et al. 2013), generally demonstrating favorable effects across a broad range of conditions. Management-centered assessments of ES-provision related resilience properties however are scarce in that context: As an exception Knoke et al. (2023) investigated economic resilience aspects, demonstrating that stand management promoting regeneration within gaps significantly reduces post-disturbance recovery times, while even slightly surpassing the clear-cut baseline in absolute economic performance. An important caveat of their research is, however, that the entire tree regeneration is assumed to survive disturbances, an assumption which is likely overly optimistic. Besides that, they focus their assessment on exemplary disturbance events, precisely controlling the pre-disturbance stand state. Despite gaining valuable insight, such a procedure is unable to quantify resilience in the face of complex, reoccurring disturbance regimes. Finally, they follow a simulation-free closed-form approach, making it difficult to adapt their procedure to other potential resilience-enhancing strategies. We thus improve their assessment framework:

(i) by exposing regeneration cohorts to the disturbance regime,
(ii) by replacing the closed-form approach with a simulation-based assessment, allowing us to assess the compound, probability-weighted effect and
(iii) by including aboveground carbon sequestration as second ES besides financial returns.

## Methods

**Fig. 1:**
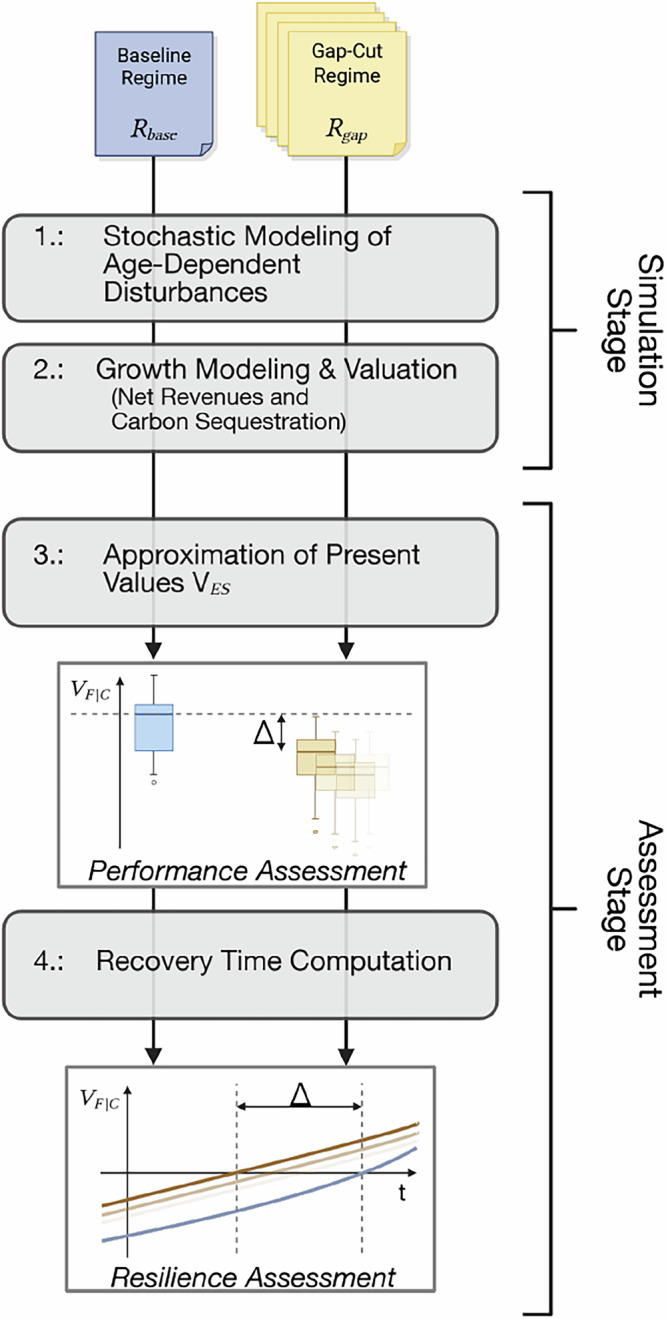
Overview of the essential modeling and assessment procedure. Details are provided in the main text.

Figure 1 gives a brief overview of our assessment procedure, comparing multiple gap-cut regimes (*R*_*gap*_) with a clear-cut baseline (*R*_*base*_). We define the regimes as simple management rules: Under *R*_*base*_, stands are established on bare soil, thinned according to a yield-table growth model (Section “Modeling growth and yield”), and harvested at rotation age followed by immediate reforestation, a procedure that results in an even-aged management cycle. In each *R*_*gap*_ however, a prescribed gap cut at stand age *age*_*gap*_ removes a share *s*_*gap*_ of the tree canopy in advance of the stand rotation age. This creates suitable growing conditions for regeneration prior to the final harvest of the whole stand, thus enabling a new tree cohort to grow. Both cohorts – the remaining original stand as well as the former gap-cut regeneration – are subsequently thinned according to the yield-table until their individual harvest at reach rotation age. Reforestation of a cohort takes place immediately after its harvest.

To generate ecosystem-service (*ES*) time series, we subject each regime to a stochastic, age-sensitive disturbance model (Fig. 1, Step 1, see Section “Simulating ES-provision time series”). Growth, yield, and valuation models then translate the outputs into provision streams of (a) net revenues and (b) aboveground carbon sequestration (Step 2). In the assessment stage, these provision streams are converted into financial value (*V*_*F*_) or sequestration value (*V*_*C*_) series, which refer to the cumulated future production potential of both *ES* (Step 3). Their post-disturbance recovery time (*t*_*recov*_) computed in Step 4 serves as central resilience metric. Comparing *t*_*recov*_ across *R*_*gap*_ and *R*_*base*_ quantifies the resilience effects of gap cuts with respect to both investigated *ES*, while analyzing the distributions of *V*_*C*_ and *V*_*F*_ enables performance evaluation and trade-off detection.

The entire simulation and analysis procedure was implemented in R (R Core Team, 2024), parameterized to represent average central German conditions for growth, disturbance regimes, and wood prices (Section “Simulating ES-provision time series”). We focus on pure Norway spruce (*Picea abies* KARST.) stands, though the framework itself is generalizable to other species and mixtures.

The following sections present the theoretical foundation regarding *V*_*C*_ and *V*_*F*_ (Section “The present value rationale”), the disturbance and growth models used (Section “Simulating ES-provision time series”), the approximation of *V*_*C*_ and *V*_*F*_ from finite-length ES-provision series (Section “Approximating VES from finite length time series”), and finally the resilience and performance assessment (Section “Analyzing gains in recovery speed and associated trade-offs”).

### The present value rationale

Building on Knoke et al. (2023), we use the post-disturbance recovery time of the present value *V* as central resilience metric for the assessed *ES*. Regarding financial returns, we index *V* with *F*, and for carbon sequestration with *C*. Both *V*_*F*_ and *V*_*C*_ imply that a stand fulfills the Markov property (Grimmett and Stirzaker, 2001, p. 213–214), i.e., transitions depend only on the current stand state, which must thus encode all factors relevant for its future development. Under this assumption, each state has one associated *V*_*F*_ and *V*_*C*_, defined as the expected sum of discounted future provisions over an infinite horizon (Equation 1). This approach is standard in dynamic optimization (Bellman 1957), while rooting also deep in forest economics since Faustmann’s soil expectation value (Johansson and Löfgren 1985).

1 $${V}_{ES}={\mathbb{E}}\left[\mathop{\sum }\limits_{t=0}^{\infty }{p}_{ES,t}\,\frac{1}{{(1+r)}^{t}}\right]$$

$$\begin{array}{lcl}\,\mathrm{where\; :} & & \\ V & & \,\mathrm{is\; the\; present\; value\; of\; future\; ES\; provisions}\,,\\ ES & & \,\mathrm{is\; the\; assessed\; Ecosystem\; Service,\; either\; financial\; revenue\; or}\\ & & \,\mathrm{aboveground\; carbon\; sequestration}\\ {p}_{ES,t} & & \,\mathrm{is\; the\; provision\; of\; the\; ES\; at\; time}\,t,\\ r & & \,\mathrm{is\; the\; applied\; discount\; rate.}\end{array}$$

*V*_*F*_ and *V*_*C*_ thus represents the equivalent one-time provision to all future provisions under discount rate *r*, given the current state. Since disturbance events make development paths stochastic, *V*_*F*_ and *V*_*C*_ are the expectations over all possible future paths, adjusting for all their individual outcomes and probabilities.

*V*_*F*_, referred to as the ‘forest value’ by Knoke et al. (2023), typically follows a sawtooth pattern in even-aged stands: increasing with stand age as thinnings and final harvest approach, peaking at harvest, and afterwards resetting to the soil expectation value. A disturbance lowers *V*_*F*_ by shifting the stand to a less desirable state, due to a complete or partial drop-out. If two stands are subjected to the same disturbance impulse, the stand with higher resilience will regain its *V*_*F*_ more quickly (Knoke et al. 2023). We capture this difference by setting a threshold *F*_*bound*_ and tracking *V*_*F*_’s recovery time towards this threshold after each simulated disturbance.

The same framework applies to non-financial *ES*, without need for prior monetization. In our case, aboveground carbon sequestration can be represented as a time-discrete provision series analogous to cash flows, and direct discounting of these non-monetary *ES* provisions is a widely accepted procedure (see for example Jarisch et al. 2022). However, the appropriate discount rate differs, as human time preferences deviate and global production trends may vary: Empirical evidence suggests non-monetary ecosystem services like carbon sequestration should be discounted about 0.9% ± 0.3 below production goods (Baumgärtner et al. 2015). We therefore apply 1.5% p.a. for net revenues, following empirical forest manager behavior in central Germany (Möhring 2001), and 0.5% p.a. for aboveground carbon sequestration.

The infinite horizon in Equation 1 is intractable computationally and can only be realized in closed-form analytic approaches. But as discounting rapidly diminishes far-future provisions, truncating the horizon at 1000 years is a feasible compromise: At the rates applied, provisions beyond this point become negligible (Knoke et al. 2021). Thus, we approximate *V*_*F*_ and *V*_*C*_ by replacing infinity with a 1000-year horizon.

### Simulating ES-provision time series

To simulate time-series data for the two examined ES, we developed a time-discrete, stand-level disturbance and growth model. Its core is a stochastic simulation of age-dependent survival of tree cohorts, defined as age-homogeneous stand portions. In a gap-cut regime, the stand is established as even-aged (one cohort) until a specified share *s*_*gap*_ is harvested at *age*_*gap*_. This gap cut establishes another cohort, and both grow in parallel until one reaches rotation age or is lost to disturbance, triggering immediate replanting. The baseline regime *R*_*base*_ is modeled as a special case without gap cuts, leaving the stand even-aged throughout. Each *R*_*gap*_ is defined by a unique, constant setting regarding *s*_*gap*_ and *age*_*gap*_: Regarding *s*_*gap*_, we tested settings of 0.1, 0.15, 0.2, 0.25 and 0.3, while for *age*_*gap*_, we choose 20, 40 and 60 years. Including all possible combinations, this resulted in 15 unique gap-cut regimes, each with an individual combination of gap size and timing.

For *R*_*base*_, we apply a fixed rotation age *u* = 85 years, at which the stand is harvested entirely. This represents a compromise between empirically common rotations of 100 + years and considerably shorter recommendations under climate change (e.g., Knoke et al. 2021), which are rarely fully adapted so far in practice. The same is applied in all gap-cut regimes, once a cohort reaches *u*.

#### Modeling age-dependent survival

**Fig. 2:**
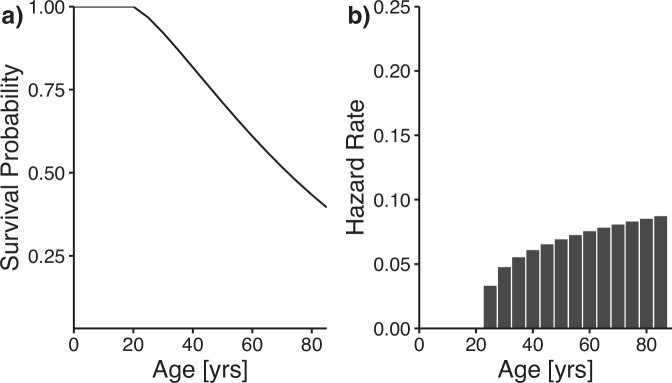
Age-dependent survival probabilities (Plot **a**, see Brandl et al. 2020) and the derived discrete hazard rates (**b**).

Stochastic, age-dependent disturbance events were modeled using survival functions from Brandl et al. (2020), which describe the probability of a stand reaching a given age without disturbance under specified climatic conditions. For pure Norway spruce stands, these functions are parameterized by the average temperature of the warmest month (${\bar{T}}_{w}$) and precipitation in the warmest quarter (∑*P*_*wQ*_). We set ${\bar{{\rm{T}}}}_{{\rm{w}}}=27.4\,^\circ {\rm{C}}$ and ∑*P*_*wQ*_ = 176mm, based on an RCP 8.5 (Riahi et al. 2011) climate projection for the central German uplands (Lat: 51.268720, Long: 9.816694) using BCC-CSM1-1 (Wu et al. 2014). Survival probabilities were converted into hazard rates (in our case 5-year-step failure probabilities) following Staupendahl and Möhring (2011). Both are given in Fig. 2.

To determine the survival or failure of a given cohort, we compared its age-dependent hazard rate (*hz*) to a random number *y* ~ *U*(0, 1) drawn at each simulated time step for the whole stand. Cohorts survived if *y* > *hz*; otherwise, we simulated a complete drop-out of the cohort with subsequent salvage logging and immediate replanting. Random seeds were varied between simulation runs but held constant across the regimes within one run, ensuring that stands managed under *R*_*gap*_ and *R*_*base*_ were exposed to identical disturbance impulses. This provides counterfactual comparability: Each disturbance simulated in *R*_*base*_ has a direct analog in each gap-cut simulation, thus illuminating how a given disturbance would have played out *if* the stand would have been (and will be) managed under a given gap-cut regime instead.

With storms as main disturbance driver in central European forests (Schelhaas et al. 2003), and vulnerability to storms primarily linked to tree height (Schmidt et al. 2010), the survival functions of Brandl et al. (2020), derived for even-aged stands, likely overestimate mortality in uneven-aged conditions. In our setting, a young cohort resulting from a prior gap cut is influenced by taller neighbors, thus showing reduced height growth for its given age (cf. Section “Modeling growth and yield”). Using unadjusted hazard rates would therefore likely introduce a bias against gap-cut regimes. We correct this by applying a reduction factor *red*_*hz*_ to non-leading cohorts, such that

2 $$h{z}_{{\rm{new}}}=re{d}_{hz}\cdot hz,\,0\le re{d}_{hz}\le 1$$

The reduction increases with the age difference (*Δage*) between the older and younger cohort, and is stronger for smaller cohorts, resulting from a lower setting of *s*_*gap*_, which face slower height growth and thus less wind exposure than larger ones.

Empirical data on these effects is, to our knowledge, absent, so we adopt simple linear assumptions (Supplementary A). Within leading cohorts, gap edges may increase wind loading temporarily (Panferov and Sogachev 2008). However, we can assume this effect to be offset by longer-term stability gains of the edge trees, resulting from more favorable height/diameter relations (Bošeľa et al. 2014), rendering those trees even more stable a few years after the gap cut. We assume both effects to approximately cancel each other out, thus applying no reduction in leading cohorts.

#### Modeling growth and yield

The above-described model elements – gap-cut regimes and stochastic age- and stand-structure dependent survival – allow for quick and efficient sampling of possible development-paths of a stand system managed under a specific regime. We refer to these paths as *trajectories*, each containing information regarding a stand’s age composition and potential disturbances at each simulated time step.

For each trajectory, growth and yield are modeled ex-post using functions from a recently published yield table framework (Albert et al. 2022; Nuske et al. 2022), which represent current growth dynamics in north-western and central Germany. We parameterized those for average central German conditions, yielding a site index of 21.9 m (mean stand height at age 50) for even-aged stands. The yield table framework contains information on standing volume and average diameters at breast height (DBH), but omits volume estimates for stands younger than 15 years due to its designated use in forest management planning. We filled these gaps via linear interpolation to ensure coverage of young stand ages in subsequent carbon sequestration calculations (Section “Estimating ecosystem services”). The yield-table framework builds on a state-of-the-art thinning regime and includes resulting volume yields and corresponding average DBHs, assuming rather progressive thinnings in low age classes, which are gradually reduced with increasing stand height.

Assuming no growth interaction among neighboring trees of different age, trajectories combined with yield table data would suffice to derive standing and harvested volumes (including DBH). To account for competition changes, however, we adjust cohort-level growth: Gap regeneration is assumed to grow more slowly under reduced light and nutrients, while older trees benefit from decreased stand density. We assume suppression effects to be stronger for small, young gap cohorts in older stands and weaker for larger gaps in younger stands (see Supplement B). Conversely, the leading cohort is assumed to undergo temporary growth acceleration following a gap cut, as canopy opening improves growing conditions, particularly near gap edges. The magnitude of this effect depends on relative gap size *s*_*gap*_, with further details provided in Supplement C.

#### Estimating ecosystem services

##### Net revenues

The resulting growth information, including removed volumes with the associated DBH- and disturbance-information, was used to estimate net revenues from wood sales and planting costs. For each time step *t*, we calculated the cash flow *CF* resulting from silvicultural measures as the sum of net revenues from thinnings *NR*_*thin*_, final harvests *NR*_*harv*_, gap cuttings *NR*_*gap*_ and salvage loggings *NR*_*salv*_, reduced by all planting costs *C*_*plant*_ occurring at *t* across all *n* present cohorts *C* (Equation 3).

3 $$C{F}_{t}=\mathop{\sum }\limits_{C=1}^{C=n}N{R}_{thin,t}+\mathop{\sum }\limits_{C=1}^{C=n}N{R}_{harv,t}+\mathop{\sum }\limits_{C=1}^{C=n}N{R}_{gap,t}+\mathop{\sum }\limits_{C=1}^{C=n}N{R}_{salv,t}-\mathop{\sum }\limits_{C=1}^{C=n}{C}_{plant,t}$$

Net revenues were estimated from volume and DBH data produced by the growth simulations, using the R-package *woodValuationDE* (Fuchs et al. 2023), which builds on DBH-dependent revenue and cost functions from von Bodelschwingh (2018). We assumed average wood quality, full accessibility for standard harvesting, and applied a mix of fully mechanized techniques for small DBH and motor-manual procedures for large DBH (see Fuchs et al. 2023 for details). A baseline price of 92.47 € per cubic meter over bark was used for the reference assortment, representing average prices obtained within the public forest administration in the central German federal state of Hesse between 2010 and 2015 (von Bodelschwingh, 2018).

Regarding salvage loggings, *woodValuationDE* provides factors to decrease the above-mentioned baseline price as well as increasing the associated logging costs. Based on Fuchs et al. (2022), we opt for lowering the baseline price to 74%, while increasing associated logging costs to 115%. These factors are empirically backed for disturbance events causing moderate damages in wood quality and a temporary regional oversupply (Fuchs et al. 2023).

Reforestation was assumed immediately after a final dropout of a cohort, regardless of whether it followed scheduled harvests or salvage logging. Fixed planting costs of 2000 € ha^−1^ were applied after regular harvests, assuming no extensive soil preparation and medium plant densities (Ministry of Agriculture in Lower Saxonia, 2019). These costs were doubled after salvage loggings to reflect higher costs for material, labor, and more challenging site conditions. Regarding the regeneration occurring in gap cuts we reduced the original costs by 50%, assuming that natural regeneration occurs in sufficient abundance to make artificial planting obsolete on at least half of the concerned area.

##### Aboveground carbon sequestration

Annual aboveground carbon sequestration was derived from the annual volume growth over each time step. We converted those volumes to dry masses using the mean of all entries in an open-access wood density database (Carsan et al. 2012), resulting in 410 kg m^−3^. Assuming an average carbon content of 0.5 kg_c_ kg^−1^ (dry weight) (Sandström et al. 2007), we obtained a conversion factor of 205 kg_c_ m^−3^, which was applied to transform the annual volume growth into aboveground carbon sequestration estimates.

### Approximating *V*_*ES*_ from finite length time series

**Fig. 3:**
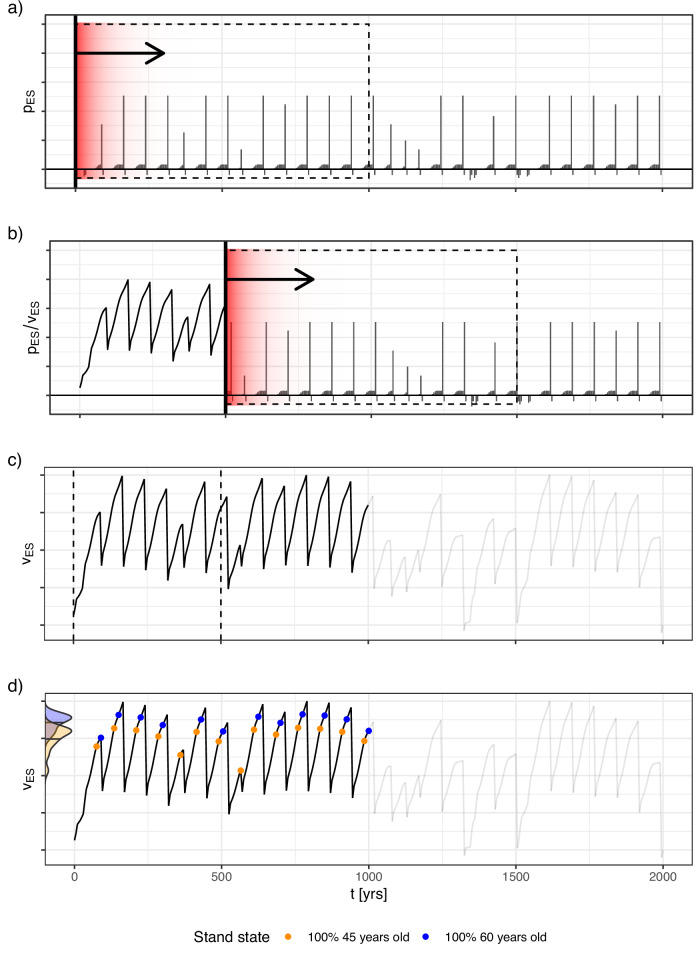
The moving-window approach used to approximate the present values of ecosystem services *V*_*ES*_ per stand state based on the simulated ES-provision time series. **a** Gives an example of a simulated provision time series, illustrating the moving window used to compute the *V*_*ES*_ samples. We repeat this computation for each time step, rolling the moving window along the simulated time series (**b**). As soon as the moving window overshoots the end of the series, the process is stopped and the time series is truncated (**c**). We then proceed with aggregating the samples of *V*_*ES*_ per state, two exemplary aggregations are illustrated in panel (**d**). Detailed explanations provided in the main text

As described in Section “The present value rationale”, we opted for the present value *V*_*F*_ or *V*_*C*_ of a stand and its post-disturbance recovery-time as key metric for the resilience assessment. Instead of deriving *V*_*F*_ and *V*_*C*_ of a given stand under a defined regime in a closed, analytic form, as for example done by Knoke et al. (2023), we estimated both by means of a Monte-Carlo simulation (MCS). This estimation process is visualized for *V*_*F*_ in Fig. 3.

From a stakeholder’s perspective, a forest stand can be perceived as an asset generating streams of *ES* provisions, and the disturbance- and growth model and the connected valuation module described above enables the quick simulation of those streams (Fig. 3a). At each time step, the resulting provision under the applied silviculture regime was logged. If we assume a deterministic trajectory regarding future stand development – implying that the underlying trajectory is totally certain – one can approximate *V*_*F*_ of the forest stand at *t* = 0 by rolling out a 1000-year and thus sufficiently long (see Section “The present value rationale”) window, discounting all provisions within the window relative to *t* = 0, and summing up their resulting present values (see Equation 1). This results in the classical soil expectation value. One can continue the procedure by shifting a moving window further in time, repeating the calculation at each time step *t* visited (Fig. 3b). Once the end of the moving window overshoots the end of the available time series, the results become increasingly more biased as fewer and fewer values lay within the window – we circumvent this issue by truncating the resulting series of *V*_*F*_ as indicated in Fig. 3c.

In our stochastic setting however, the result computed at each step is not the *V*, but instead one sample from its underlying distribution. Many possible trajectories exist over the 1000-year horizon, each yielding a different net present value. By computing it in the way just described, one would compute the net present value associated with just that one sample trajectory this singular provision-stream happens to represent. To approximate the underlying distribution, the resulting samples need to be aggregated by their underlying stand state, i.e., the cohort composition at the specific time step. Figure 3d shows two such aggregations as an example: All data points corresponding to stand ages of 45 years (orange) resp. 60 (blue) years are selected, and the expected value for each state is estimated as the mean of the aggregated samples. As the precision depends on how often a given state is encountered in the simulated dataset, we simulated 1000 time series of 2000 years each for every regime, ensuring proficient abundance of even rare states in the dataset. Note that the intention is not to provide a literal forecast of stand development across such a time span. Instead, this simulation length serves as a methodological device: as we keep growth and management parameters constant, we approximate an infinite time horizon under static conditions, ensuring stable valuation results that are not sensitive to endpoint effects. Other than the discount rate adjustment (Section “The present value rationale”), we used an identical estimation process for *C*.

### Analyzing gains in recovery speed and associated trade-offs

After aggregation, the simulated dataset contains estimates of *V*_*F*_ and *V*_*C*_ for each stand state under each regime *R*. By re-joining these with the original trajectories, we obtain *V*-paths over time, including information on age structure (the state), the associated *V*_*F*_ and *V*_*C*_, and occurrence of disturbance events. Disturbances cause sharp declines in *V*_*F*_ and *V*_*C*_. Since disturbance events were explicitly logged in the dataset, we can distinguish them from declines due to final harvests. For each disturbance, we measure recovery time of both *V*_*F*_ and *V*_*C*_ as the period until they reach their thresholds *F*_*bound*_ and *C*_*bound*_ (see Section “The present value rationale”), defined following Knoke et al. (2023) as the average *V*_*F*_ or *V*_*C*_ under the baseline clear-cut regime. Due to the time-discrete nature of the underlying disturbance model, the precise time at which *V* equals its threshold cannot be measured directly, instead we estimate it by interpolating linearly between the two closest data points.

Our counterfactual disturbance simulation (Section “Modeling age-dependent survival”) creates one-to-one matches between disturbance events in *R*_*base*_ and each *R*_*gap*_. This pair-wise setup enables comparison of recovery times at the level of individual disturbance events. From this, we derive: (a) average recovery-time changes for each *R*_*gap*_ relative to the clear-cut baseline and (b) the distribution of those changes. We interpret (a) as the expected resilience gain of a given gap-cut regime and (b) as the variability of this change. As we also track the pre- and post-disturbance stand state, disturbances can be categorized into the following categories:

1. Disturbances affecting a stand prior to any gap-cut and thus without any regeneration present, resulting in a complete failure of the stand;
2. Disturbances affecting a stand after a gap-cut, thus with regeneration present, but still resulting in a complete failure of the stand;
3. Disturbances affecting a stand after a gap-cut activity, thus with regeneration present, concerning only the older stand cohort;
4. Disturbances replacing a former gap cohort that became the leading cohort due to failure or harvest of the formerly leading cohort.^1^

Our performance assessment and subsequent trade-off analysis is based on the soil expectation value (*SEV*) of a given *ES*, which can be interpreted as the production potential of bare forest soil under the specific regime (Johansson and Löfgren 1985). As all our simulations start from bare forest soil, the first estimate of *V*_*F*_ resp. *V*_*C*_ equals the the *SEV*_*F*_ and *SEV*_*C*_, respectively, of that run. Thus, we can access a well-established performance measure for each *ES* by simply selecting the first estimates of *V*_*F*_ resp. *V*_*C*_, and grouping them for each silvicultural regime investigated.

We can now derive a trade-off metric *τ* for each gap-cut regime *R*_*gap*_ by dividing the average reduction in *SEV*_*F*_ and *SEV*_*C*_, respectively, of any gap-cut regime compared to the clear-cut baseline, with the recovery-time gain this regime shows relative to that clear-cut baseline:

4 $${\tau }_{{R}_{gap}}=\frac{\overline{\Delta {{\rm{P}}}_{{R}_{gap}\leftrightarrow {R}_{base}}}}{\overline{\Delta {t}_{{\rm{rec}}{R}_{gap}\leftrightarrow {R}_{base}}}}$$

$$\begin{array}{rcl}\,{\rm{where}}\; : & & \\ \tau & & \,{\rm{is}}\; {\rm{the}}\; {\rm{trade}}\mbox{-}{\rm{off}}\; {\rm{metric}},\\ \Delta \,{\rm{P}} & & \,{\rm{is}}\; {\rm{the}}\; {\rm{difference}}\; {\rm{in}}\,{\rm{SEV}}\\ {R}_{gap} & & \,{\rm{indicating}}\; {\rm{a}}\; {\rm{specific}}\; {\rm{gap}}\; {\rm{cut}}\; {\rm{regime}}\; {\rm{and}}\\ {R}_{base} & & \,{\rm{indicating}}\; {\rm{the}}\; {\rm{clear}}\mbox{-}{\rm{cut}}\; {\rm{baseline}},\; {\rm{while}}\\ \Delta {t}_{{\rm{rec}}} & & \,{\rm{is}}\; {\rm{referring}}\; {\rm{to}}\; {\rm{the}}\; {\rm{equivalent}}\; {\rm{difference}}\; {\rm{in}}\; {\rm{recovery}}\mbox{-}{\rm{time}}.\\ \end{array}$$

${\tau }_{{R}_{gap}}$ can thus be interpreted as the change in *SEV*_*C*_ or *SEV*_*F*_, that must be accepted for one year of average recovery-time reduction when applying (and indefinitely sticking to) the regime *R*_*gap*_, compared to the clear-cut baseline. This metric thus represents the opportunity costs associated with one year of average recovery-time reduction, enabling us to identify regimes that may improve resilience at low costs. If a gap-cut regime outperforms the baseline in both performance and resilience, ${\tau }_{{R}_{gap}}$ becomes non-negative, making the regime *dominant*; the regime increases resilience without negative impacts regarding the performance.

## Results

### Changes in resilience

**Fig. 4:**
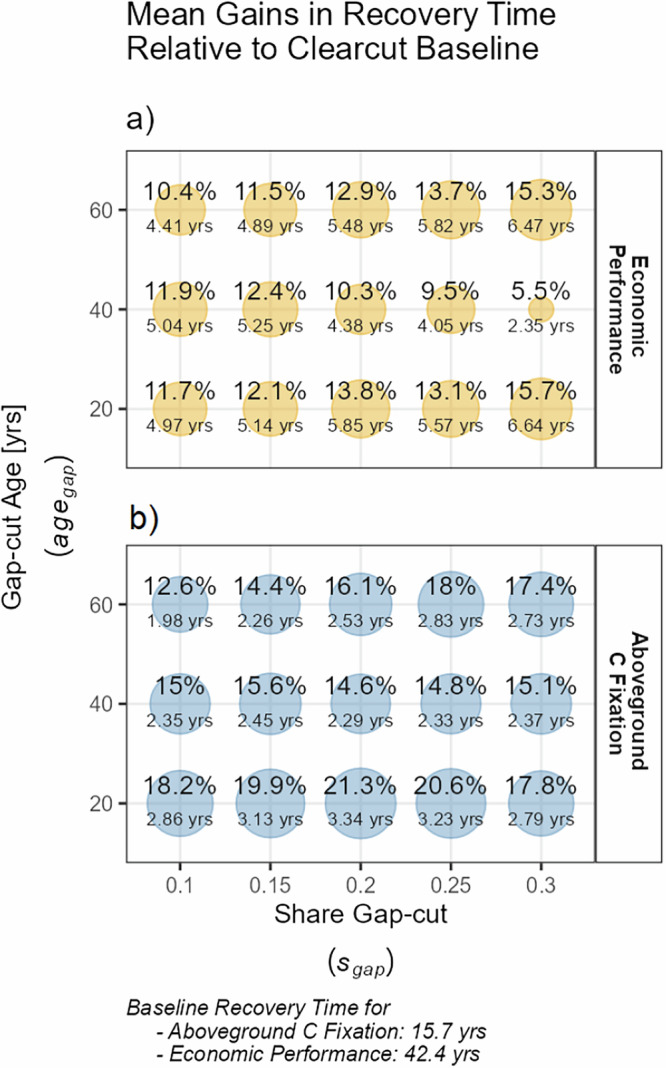
The observed average relative gains in recovery time for each investigated gap-cut regime, referring to economic performance (**a**) and aboveground carbon sequestration (**b**). Absolute values are denoted below.

The even-aged clear-cut regime used as baseline needed on average 42.4 years to reobtain the economic present value reference level. Regarding carbon sequestration, this recovery-time was considerably shorter, amounting to 15.7 years on average. Concerning their average recovery times, all of the investigated gap-cut regimes were able to reduce these time spans (Fig. 4). However, the degree of reduction varied between the gap-cut regimes, depending on the accessed *ES* and their individual settings regarding *age*_*gap*_ and *s*_*gap*_. Regarding economic resilience, the recovery time of the gap-cut regimes was reduced by up to 6.6 years, which translates to a relative time saving of up to − 15.6%, compared to clear-cut regimes (Fig. 4a). Gains regarding carbon sequestration (Fig. 4b) were lower in absolute values, peaking at 3.3 years, while relative reductions exceeded the economic dimension in large parts, due to the lower recovery-time baseline. Gap-cut regimes performing the strongest resilience-wise in the financial aspect did not necessarily do so regarding carbon sequestration, and vice versa. The average net gains between the individual gap-cut regimes varied far more in the financial than in the carbon sequestration-oriented setting.

**Fig. 5:**
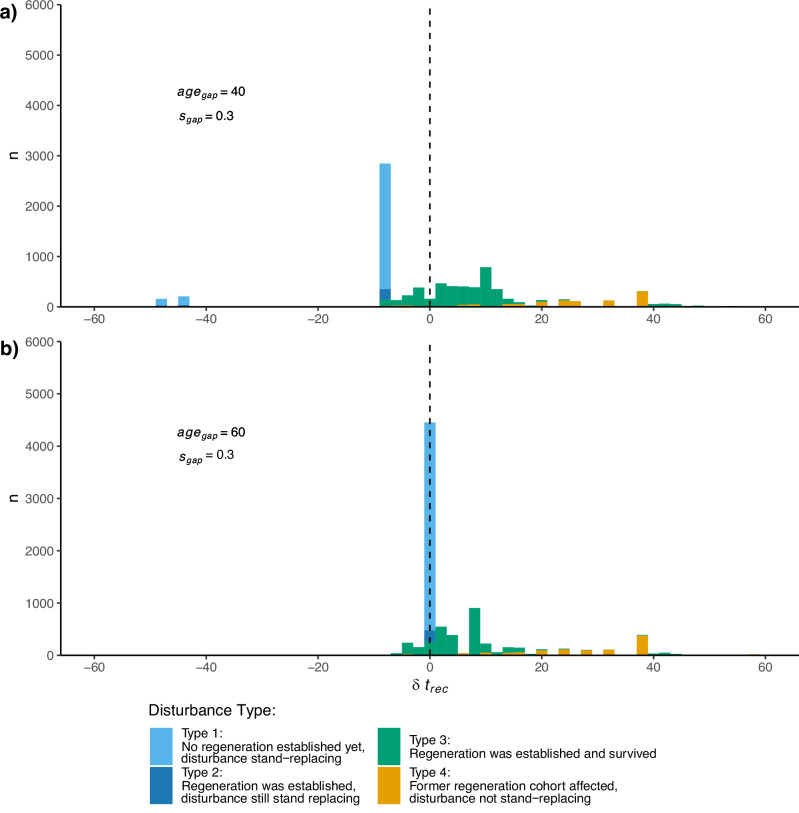
Distributions of recovery-time gains for two of the simulated gap-cut regimes: (**a**) refers to the regime involving gap cuts of 30% at age 40, while (**b**) refers to the regime with same-sized gap cuts at age 60. A negative recovery-time gain refers to a net elongation of the recovery process relative to the same disturbance event in the clear-cut baseline. Colors refer to the disturbance type, taking into account the stand state before and after the disturbance (for details see the list in Section “Changes in resilience”). We refer to the Supplementary E for the distributions of other gap-cut regimes than the two presented here.

Figure 5 illustrates the influence the 4 different disturbance types (see Section “Analyzing gains in recovery speed and associated trade-offs”) exert on the aggregated average recovery times presented in Fig. 4 by representing the distributions of recovery-time gains, as well as the disturbance-types the individual observations resulted from. For the sake of clarity, we focus on two gap-cut regimes and the financial setting only. The annotations denote the corresponding gap-cut related parameters.^2^ For both gap-cut regimes, substantial recovery time gains (relative to their clear-cut baseline disturbance-counterparts) only occurred for disturbances of type 3 and 4. Stand-replacing disturbances of type 1 and 2, on the other hand, resulted in no net recovery time gains (Fig. 5a) or even net elongations of the recovery process (Fig. 5b). Thus, the net-positive recovery-time gains visible in Fig. 4 are, at least for the regimes depicted in Fig. 5, driven by the non-stand replacing disturbance cases. While disturbances of type 1 and 2 show a low variance in their attributed recovery-time gains (or, in case of Fig. 5b, recovery-time elongations), gains attributed to the partial distribution types 3 and 4 tend to be spread much wider.

### Performance and trade-offs

**Fig. 6:**
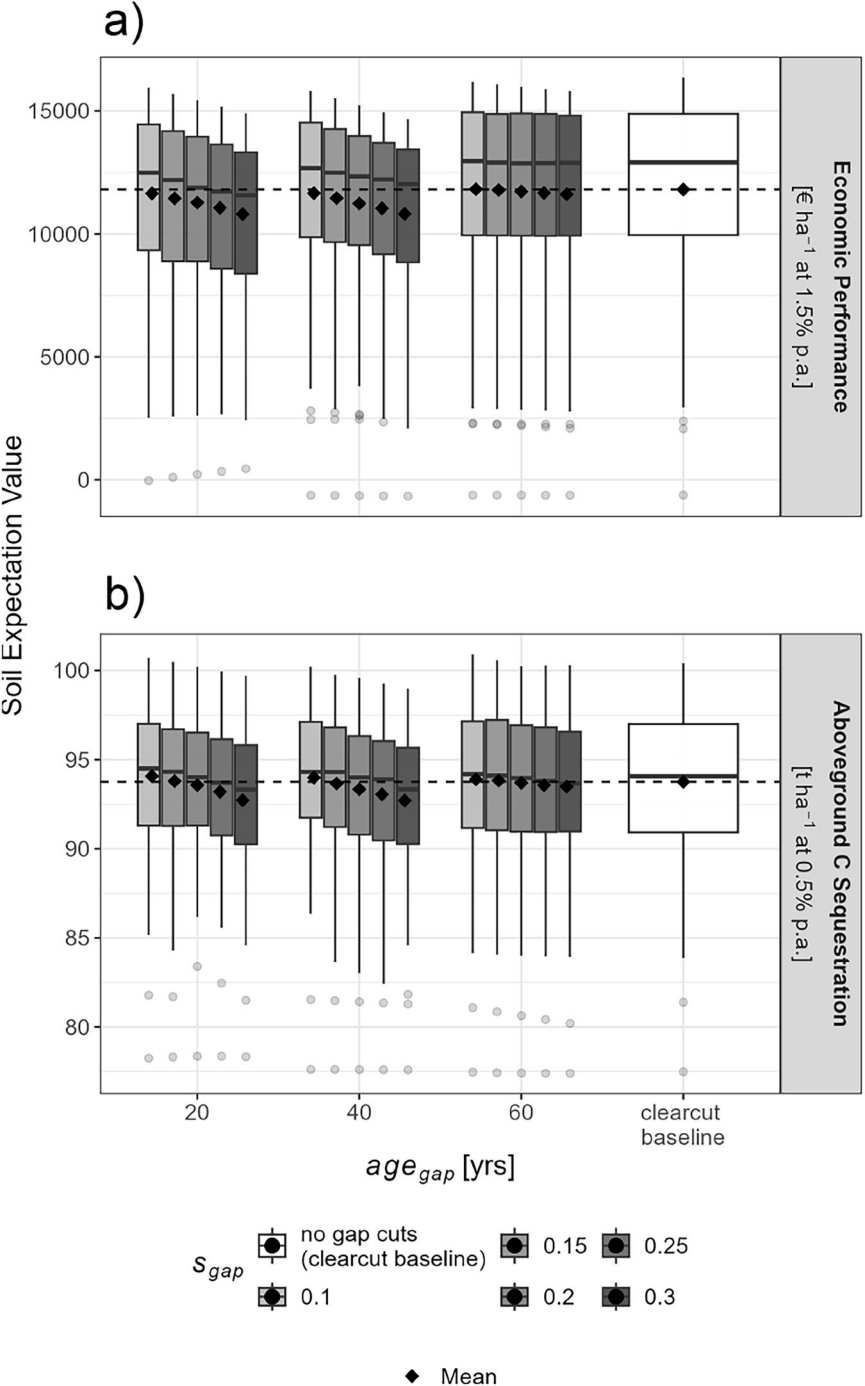
Achieved distributions of soil expectation values for the investigated regimes, for both economic performance (**a**) and aboveground carbon sequestration (**b**). The dashed line marks the mean soil expectation values obtained by the clear-cut baseline regime, whose distributions are shown on the right.

When measuring the performance of the regimes applied using their obtained SEVs, only some of the investigated gap-cut regimes were able to break-even with the clear-cut baseline, with only a few of them even surpassing it slightly. However, trends vary depending on the accessed ES (Fig. 6).

#### Economic performance

Focusing on financial provisions, the clear-cut baseline yielded an average *SEV*_*F*_ of 11, 793 € ha^−1^ (Fig. 6a). This performance was not matched by most and only marginally exceeded by the two most profitable gap-cut regimes, with the best surpassing the baseline by 36 € ha^−1^, involving gap cuts of 10% at age 60. In contrast, underperformance in less productive regimes was more pronounced, with average shortfalls of up to 936 € ha^−1^ (−7.9%) under 30% cuts at age 40.

#### Aboveground carbon sequestration

Compared to the financial context, more gap-cut regimes matched the clear-cut baseline in the carbon sequestration domain regarding the obtained *SEV*_*C*_ (Fig. 6b). This over-performance was particular pronounced for regimes involving smaller gap cuts (*g*_*size*_ = 0.10 to 0.15), which obtained a higher SEV for all investigated *age*_*gap*_. Regimes with larger *g*_*size*_ obtained lower carbon-related SEVs than the clearut baseline, which obtained an SEV of 93.7 *t*_*C*_ ha^−1^.

#### Trade-offs

Most of the investigated gap-cut regimes increase resilience at the cost of SEV reduction, indicating a trade-off between resilience and SEV (both in economic terms and carbon sequestration, Table 1). Negative values for *τ* (i.e., increasing resilience at the cost of bare forest soil related performance) occurred more often in the financial than in the carbon sequestration domain. Gap-cut regimes involving partial harvests at medium stand ages tend to perform poorer regarding their *τ* than gap-cut regimes relying on late- or early-timed partial harvests.

**Table 1:** Trade-offs, referring to the bare forest soil NPV, quantified using *τ* (see Section “Performance and trade-offs”), as well as average recovery-time gains listed for each investigated regime and indicator.

	*s*_*gap*_	*age*_*gap*_	$\overline{\Delta {t}_{{\rm{rec}}{R}_{gap}\leftrightarrow {R}_{base}}}$	${\tau }_{{R}_{gap}}$
		[yrs]	[yrs]	[(*t*_*C*_ or €) *ha*^−1^*a*^−1^]
Economic Performance	0.30	40	2.35	−398.28
	0.25	40	4.05	−183.69
	0.20	40	4.38	−125.90
	0.30	20	6.64	−116.67
	0.25	20	5.57	−103.98
	0.20	20	5.85	−69.69
	0.15	40	5.25	−69.37
	0.15	20	5.14	−48.05
	0.10	40	5.04	−36.52
	0.30	60	6.47	−22.37
	0.10	20	4.97	−19.27
	0.25	60	5.82	−15.08
	0.20	60	5.48	−7.00
	0.15	60	4.89	0.28
	0.10	60	4.41	8.22
Aboveground C Sequestration	0.30	40	2.37	−0.29
	0.25	40	2.33	−0.20
	0.30	20	2.79	−0.17
	0.20	40	2.29	−0.10
	0.25	20	3.23	−0.04
	0.15	40	2.45	−0.01
	0.30	60	2.73	0.01
	0.20	20	3.34	0.03
	0.25	60	2.83	0.03
	0.20	60	2.53	0.06
	0.10	40	2.35	0.07
	0.15	20	3.13	0.09
	0.15	60	2.26	0.09
	0.10	60	1.98	0.13
	0.10	20	2.86	0.14

Sorted by *τ*, thus starting with the most pronounced trade-off.

In total, 9 regimes increased resilience without decreases in performance in the carbon sequestration context, while only 2 regimes did so in the financial assessment. The regimes with the highest recovery-time reductions did not necessarily represent the best trade-offs, meaning their resilience-gains came in rather costly.

## Discussion

The present study sought to generalize the assessment of resilience for two ecosystem services by considering the probability of multiple resilience outcomes. To achieve this, we quantified gap-cut induced resilience changes and associated trade-offs on a stand level, using the recovery time of the present value (*V*) towards a predefined threshold as central resilience indicator. Focusing on the time a stand needs to regain a given future provision potential yields an intuitive and plausible resilience metric, relevant both from a scientific as well as from a management-centered perspective.

An important feature of the presented approach is its deliberate emphasis on parsimony. Rather than using complex, single-tree level growth models, we rely on a comparatively simple stand representation combined with a stochastic disturbance regime to capture the main impacts the investigated regimes exert on post-disturbance recovery. This level of simplicity helps to preserve transparency and interpretability, while at the same time enabling a robust quantification of resilience outcomes and associated trade-offs across management regimes and ecosystem services. At the same time, the non-closed-form nature of our approach remains open to future extensions (see Section “Prospects of further model enhancements”), including the integration of more complex growth models should additional detail be warranted. We developed this novel, simulation-based approach regarding the estimation of *V* in order to overcome the inherent limitations of analytic assessments (see Section “Comparison with previous stand-level resilience assessments”).

We demonstrated that our gap-cut regimes, despite being rather simple in design, can indeed lower post-disturbance recovery time when compared with a clear-cut-based baseline, both for financial returns and carbon sequestration (Research Question *a*). Depending on gap-size and timing, subtle performance-related trade-offs may exist, while some combinations, in particular in the carbon sequestration context, proofed to dominate the clear-cut baseline both in the resilience *and* performance assessment (Research Question *b*).

Recovery-time gain and associated trade-off patterns differ among the two investigated *ES*. In the financial context, late-timed gap cuts tend to have the lowest or even inverted opportunity costs, while these inverted costs where most pronounced with an early-timed gap-cut regime regarding carbon sequestration (Table 1). Regimes with the highest net reductions in recovery time did not necessarily show the lowest trade-offs, indicating that their high resilience gain came in rather costly. As an example, the regime *s*_*gap*_ = 0.3 ∣ *age*_*gap*_ = 20, despite showing the highest recovery-time reduction in the financial context, ranked only 12th out of 16th regarding its opportunity costs.

The positive influence many of the assessed gap-cut regimes have on stand-level resilience emerges from two different mechanisms, referred to as ‘structure’- and ‘performance’-based resilience by Knoke et al. (2023). The structural component comes to play once a pre-existing regeneration cohort survives a given disturbance and, thus, constitutes a notable value in the immediate aftermath, meaning the recovery-process of *V* starts from a higher level. As such, structural resilience depends on the presence of regeneration in the face of a disturbance, the likelihood of which depends on the stand age the gap cut is scheduled at. If scheduled at a young stand age, chances are high that regeneration is present once age-dependent disturbances occur. However, the relationship between the scheduled gap-cut age and the emerging structural resilience is more complex: If induced too early, the likelihood increases that the regeneration cohort may not survive a disturbance occurring far in the future, as its own hazard rate increases with age as well. At the same time, a more late-timed regeneration schedule decreases the lead in growth, and, if scheduled too late, the regeneration cohort might not even be established yet, diminishing any potential gains emerging from structural resilience.

The performance-based resilience component on the other side emerges from advantages over the clear-cut baseline in the performance dimension. As shown in Section “Performance and trade-offs”, some of the investigated gap-cut regimes marginally outperform or at least break even with the clear-cut baseline regarding their *SEV*. A higher *SEV* is beneficial for stand-level resilience within our recovery-time perspective, as the *SEV* acts as the starting point of the recovery process of *V* – or as a lower boundary *V* cannot fall below: As stand-replacing disturbances of type 1 and 2 (see Section “Changes in resilience”) reset *V* to its bare forest soil default, a higher *SEV* benefits a shorter recovery-process for these disturbance types. Figure 5, comparing the recovery time gains and losses of two selected gap-cut regimes, illustrates the influence of these two different resilience components on the net recovery time gain. The first regime (Fig. 5a), is characterized by a higher degree of performance-based resilience than the second regime (Fig. 5b), as the recovery time of stand-replacing disturbances (light and dark blue) is on a par with the clear-cut baseline. The same stand-replacing disturbance types tend to show net elongations regarding their recovery time in the second regime, due to considerably lower soil expectation values, i.e., lower performance. However, the second regime can at least partially compensate this drawback via the structural resilience component, manifesting itself in a lower overall proportion of stand-replacing disturbances due to the lower gap-cut establishment age. In this particular case, the increased structural resilience is not enough to fully compensate the disadvantage regarding the performance-related component, as visible in the remaining differences regarding the net recovery time gains (Fig. 4).

### Comparison with previous stand-level resilience assessments

Resilience assessments beyond the individual-tree level form a steadily growing field of research. Some approaches focus on structural aspects of the assessed stand system, as for instance Bryant et al. (2019), who aggregate several structural metrics into a scaled resilience score, grading the resilience-properties of a given stand state. As long as the aggregated indicators are indeed correlated to post-disturbance system behavior, such an assessment is valid for ranking, for instance, different stand types at a local scale. However, such a score-based approach is inherently limited on the study area it is designed for, as the disturbance drivers in other settings will differ, demanding the aggregation of different stand metrics and subsequently hindering comparability. Another issue is *which* structural indicators to choose and *how* to aggregate them, e.g., regarding their individual weighting. Other authors restrict their resilience-assessment purely on performance-related measures, as for instance Hahn et al. (2021), who quantify the economic resilience-value of different management regimes as their difference in the net present value achievable under risk. This procedure is analog to our performance-assessment (Section “Analyzing gains in recovery speed and associated trade-offs”). We would argue it does not address resilience in the sense of post-disturbance system behavior, but rather evaluates performance under a given disturbance regime. Similar as in our setting, Seidl et al. (2017) assess resilience in pure Norway spruce stands, focusing on the recovery time of the basal area following thinnings of various intensities. The metric is chosen as growing stock is strongly correlated with carbon storage, however, focusing solely on stock values (regardless if in a carbon- or financially oriented setting) is ignoring future provision flows of the *ES* in question, which are essential in a management context.

Besides Knoke et al. (2023), we are not aware of other studies utilizing the present value of future *ES* provisions and its post-disturbance recovery time as resilience metric, despite the fundamental logic being rather appealing in a management-oriented setting. Measuring *how quickly a given system regains its capacity to generate ES provisions* after a disturbance translates complex, long-term *ES* flows into a single, intuitive measure of resilience. Unlike isolated flow metrics, the recovery time of *V* captures both the speed and the integrative depth of system recovery, while including the central aspect of time preference. While Knoke et al. (2023) introduced this metric in a closed-form setting, challenging the adaption in different contexts, we generate future provision development paths from simulations. We are convinced that this approach constitutes a valuable contribution to the field. As long as data for parametrization exists and the description of the forest state can be kept simple enough, the simulation stage (Fig. 1) can be altered in order to represent different management strategies, tree species compositions and disturbance regimes. This allows a direct comparison, for example when evaluating different resilience-enhancing approaches (e.g., gap-cut induced regeneration vs. tree species admixture, tree species admixture vs. rotation age reduction, etc.). The disturbance- and growth model may also be tailored to represent different study areas, involving other disturbance agents. These changes are easier to implement than in the original closed-form setting utilized by Knoke et al. (2023), due to our strict separation of simulation and resilience assessment. We see this non-analytic, Monte-Carlo based and, thus, rather flexible and adaptable approach for deriving present value estimates as a major contribution of our study, allowing quantification of the overall effect on resilience, as probabilities for specific stand states are implicitly accounted for.

Compared to Knoke et al. (2023), who observed financial recovery-time reductions of up to 17 years, we obtained more general results by considering probabilities of resilience outcomes. Our observed recovery time reductions may seem rather low, amounting to 6.64 years in the carbon and 3.34 years in the financial context. However, one has to keep some key differences in mind when comparing these values.

First, we refer to *average* recovery time reductions across all disturbances ever recorded during the simulations, while Knoke et al. (2023) derived recovery time gains analytically for pre-defined pre-disturbance states. Their procedure generates valuable insights regarding resilience in the face of precisely defined disturbances in certain stand settings, but the resulting values cannot be directly compared to the ones presented here. As we averaged the recovery times across all occurring disturbance events, which we did not set a priori, but instead simulated stochastically based on empirical hazard rates (Section “Modeling age-dependent survival”), our values need to be interpreted as *average recovery times* instead. Not linked to specified disturbances, but rather to the expected disturbance pattern of the given stand system as a whole, we would argue that those average values may be more suited for a *general* resilience assessment. This is corroborated by the above-mentioned discussion that a simulation-based approach such as the one presented here may be more flexible to adapt for different contexts: As long as the underlying state description can be kept simple enough, the simulation stage (Fig. 1) can be adapted without altering the subsequent assessment procedure.

Second, Knoke et al. (2023) assumed a guaranteed survival of the trees in the regeneration cohort, an optimistic assumption which does not always hold true in reality. While a reduction in exposure, particular with respect to storm-induced disturbances, is plausible and backed by empirical data^3^, the assumption of guaranteed survival may render their gap-cut regime too positive, affecting both the resilience and the performance dimension.

Lastly, one has to keep in mind that our gap-cut regimes are quite simple in design, specifying only size and timing of a singular gap cut, while limiting the total amount of stand cohorts to just two. Knoke et al. (2023) in contrast deploy a refined optimization algorithm fine-tuning the harvest schedule of their gap-cut regime, resulting in a close-to-nature, continuous-cover stand management regime, outperforming their clear-cut baseline by 8% in economic performance at a discount rate of 2.5%—a performance-advantage we are not able to reach, as the complexity of our stand-management regimes is methodologically restricted by our post-MCS aggregation procedure (see Section “Prospects of further model enhancements”). However, it is worth noting that even gap-cut systems as simplistic and easy-to-implement as the ones tested within this study may decrease the post-disturbance recovery-time with little to no associated performance losses when focusing on a bare forest soil reference state. For the future, however, it would be interesting to represent the consequences of gap cut regimes using a process-based forest growth model (see Section “Prospects of further model enhancements”).

### Implications for forest management

The favorable impact on stand-level resilience indicated by our results emphasizes the relevance of gap-cut regimes which classify as uneven-aged forest management in the face of intensifying disturbance regimes. Our simulation-based assessment is in line with previous empirical studies comparing disturbance and stability characteristics of even and uneven aged systems (e.g., Mohr et al. 2024; Nevalainen, 2017; Sharma et al. 2016), corroborating the validity the results.

From a practical perspective, the advisable choice of gap size and timing will depend on the specific objectives and risk attitudes of the decision maker. A manager primarily interested in financial performance may prefer late-timed regimes with smaller gap sizes, as these tend to improve resilience without associated opportunity costs. As a benefit, those regimes tend to show favorable behavior in the carbon sequestration context as well. If financial resilience is prioritized over pure performance, one may opt for larger, yet still late-timed gap cuts, or early-timed gap cuts instead. Large mid-timed gap cuts on the other hand should be avoided, as these tend to combine poor average recovery-time reductions combined with the most severe performance trade-offs.

From the perspective of large forest property owners, the relevance of stand-level resilience might be less pronounced, as disturbances in one stand can be offset by the performance of other stands. If risk cannot be diversified spatially, however, as for instance in the case of small-scale forest ownership, stand resilience might be of greater relevance. The presented study indicates regeneration inducing gap-cuts as one feasible option to reduce disturbance risks with low associated costs.

### Prospects of further model enhancements

In its current implementation, our simulation approach is able to generate insight in the resilience and performance dimension of pure-species stands, managed under various silviculture regimes. The parsimonious growth model developed and used for this study (see Section “Simulating ES-provision time series”) ensures a high degree of transparency and interpretability, which we believe to be vital when introducing a novel quantification approach. However, the current growth model also limits associated resilience assessments (a) to forest stands small in size and (b) to management regimes simple in design.

Regarding (a), the approach used to model disturbances (see Section “Simulating ES-provision time series”), also backed by empirical data (Brandl et al. 2020), has some inherent limitations regarding the considered stand size. The current cohort-replacing mode of either undisturbed survival or total drop-out of the whole affected cohort seems to be a reasonable approximation for small-scale forest stands. Senf and Seidl (2020) obtained an average disturbance patch size of 1.09 ha across Europe, based on remote sensing, with a range between the 1^*st*^ and 99^*th*^ percentile of 0.18–10.10 ha. Thus, a strict all-or-nothing disturbance assumption within small stands can be justified. However, the approach loses credibility when increasing the total stand size, as partial disturbances become more likely. Empirical data to model those partial disturbances on a stand level is scarce, because it depends on temporal and spatial disturbance interactions as well as human interventions. For instance, in Norway spruce stands disturbance patterns depend to a large extend on the response speed of the forest manager, as a faster response can help to mitigate ongoing bark beetle outbreaks. Representing larger stands or forest landscapes in the presented model would, therefore, require a more advanced mode of disturbance simulation, which is an active field of research.

Regarding (b), with our *V* estimation process being centered around the aggregation of individual forest-value samples on identical stand states (see Section “Approximating VES from finite length time series”), the feasible complexity of the growth and disturbance simulation as well as the complexity of the applied management regimes, are inherently limited. As the forest-value estimation process demands for discrete state descriptions, as well as an overseeable amount of feasible stand states in total to guarantee sufficient sampling of the underlying distributions, growth models of higher order, i.e., single-tree based approaches, are currently challenging to integrate. In our current implementation, the state of a forest stand (and thus the reference point for any forest value) is described as a combination of only a few features, namely the age and proportion of the old growth cohort, age and proportion of the gap-cut cohort (if existing), and the silviculture regime applied. As we do not allow for only partial disturbances of any cohorts, limit the gap size to a fixed value within each regime, and simulate the stand development in discrete 5-year steps, the total amount of accessible stand states is limited. This limitation is necessary in order to ensure that each feasible state is visited often enough during the simulation process, a condition necessary to guarantee an accurate, unbiased estimation of the corresponding state- and forest-value pairs.

If more variables need to be included in the state description, for instance, to account for multiple species in a mixed stand, changes in growth conditions or timber prices, or even for investigating more complex and refined silvicultural strategies within single-tree based growth simulators, the state description inevitably becomes more complex and the accessible state space grows super-linear in size. The resulting problem of mapping expectation values of forward-looking, discounted provisions or ‘rewards’ to detailed state descriptions too complex to list and sample directly is common in machine learning, in particular in the field of reinforcement learning (Sutton and Barto, 2018), where it is commonly circumvented with the use of function approximators. Once sufficiently trained, i.e., incoporating enough samples, these approximators, typically implemented as neural networks, are able to generalize from known to unknown state descriptions, i.e., from a known stand state to a slightly altered, previously unseen one. Current examples for implementations in different, yet high-dimensional, stochastic forestry settings include Malo et al. (2021) and Tahvonen et al. (2022), illustrating the effectiveness of neural networks in the prescribed context. Deploying a neural network for state-value-mapping could enable the integration of more refined, i.e., spatially explicit, single-tree based growth and disturbance models in the resilience assessment. Such an approach would also allow testing more refined silvicultural regimes, up to the extent where even real-world strategies could be tested and compared, for instance, in process-based simulators.

## Conclusion

We tested whether or not simple, regeneration-inducing gap-cut regimes can promote stand-level resilience when compared to a clear-cut baseline regime and, if so, what the associated performance-related trade-offs are. We conclude, that:

1. Refined Monte-Carlo simulations allow generalizing stand-level resilience assessments, while retaining a higher flexibility than previous closed-form approaches.
2. Pre-rotation gap cuts to establish regeneration cohorts generally reduce post-disturbance recovery times, across various spatial and temporal settings.
3. However, pre-rotation gap cuts often reduce financial performance.
4. Even simulating simple silvicultural strategies allows demonstrating the favorable resilience-related effects of pre-rotation gap cuts.
5. In the future, we recommend coupling mechanistic growth model outcomes with forest value function approximators, to predict management impacts on ES performance and resilience.

## Supplementary information

Supplementary Information

## Data Availability

Data used in this study is available through the corresponding author.

## Footnotes

1 For further details regarding that case we refer to Supplementary D.

2 Identical histograms for the other investigated regimes as well as for the carbon sequestration setting are given in the Supplementary E.

3 Mohr et al. (2024) for example estimate the disturbance probability to be 31.3% lower in uneven-aged stands than in their even-aged counterparts, and the aforementioned survival functions proposed by Brandl et al. (2020) indicate lower hazard rates at younger stand ages.

## Code availability

All code and models developed during this study is available through the corresponding author on request.
