# Supplementary material for: Modeling the Effects of Gap Cuts on Stand-Level Resilience in Financial and Carbon Sequestration Contexts
Source: Environ Manage. 2026 Mar 18;76(4):122. doi: 10.1007/s00267-026-02418-z (PMC12995922; doi:10.1007/s00267-026-02418-z)
Supplement: Supplementary file 1 — Supplementary Information [file 267_2026_2418_MOESM1_ESM.pdf]

Supplementary Materials for:

Modeling the Effects of Gap Cuts on Stand-Level Resilience in Financial and Carbon Sequestration Contexts

Jonathan Fibich*, Alexander Lange, Thomas Clemen, Dominik Thom and Thomas Knoke

This supplementary file contains:

A: Visual representation of the applied hazard-rate reductions within non-leading cohorts

B: Details on the derivation of the growth reduction factors applied within non-leading cohorts

C: Details on the derivation of the growth acceleration factors applied in remaining leading cohorts

D: Details on disturbance-type 4

E: Remaining financial as well as carbon-oriented recovery-time gain distributions for all investigated regimes

A: Visual representation of the applied hazard-rate reductions within non-leading cohorts

We applied a reduction factor (red_{hz}) in order to decrease the originally purely age-dependent hazard rate within non-leading compartments. For details on the motivation and technical integration of that procedure we refer to Section

*Corresponding author: jonathan.fibich@tum.de

2.2.1 and in particular to Equation 2 of the main article – Figure 1 illustrates the linear assumptions applied in this context.

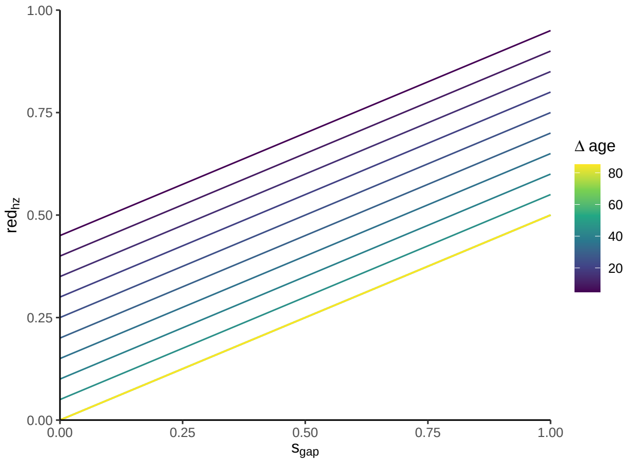

Figure 1: The applied assumptions regarding the hazard rate reduction in non-leading cohorts. Low values of red_{hz} indicate a strong discount on the original, solely age-dependent hz (see Section 2.2.1 in the main article for details). An increase in the size of the non-leading cohort (s_{gap}) yields a lower reduction, while an increase of the age difference $\Delta\ age$ between the leading and non-leading cohorts results in a higher discount of the original hz.

B: Details on the derivation of the growth reduction factors applied within non-leading cohorts

Publications quantifying the degree of growth suppression non-leading age cohorts in uneven aged stands are subjected to are surprisingly scarce. Hence, we do have to make some evidence-based assumptions in order to come up with a plausible function suitable to compute the necessary growth correction during the simulation process.

From the perspective of a sub-dominant age cohort, two parameters are central for its individual growth conditions, and, thus for the degree to which its volume growth falls behind an otherwise comparable non-suppressed counter-

part. First, the smaller the size of the gap relative to the surrounding leading cohorts, the more severe the impact is on the inner-gap growth conditions. However, gap size is not the only relevant factor, as the lead the surrounding stand has in growth matters as well, since taller trees naturally exert a higher pressure competition-wise.

Knoke and Plusczyk [2001] investigated the economic consequences of a transformation taking place from a clear-fell to a continuous-cover system, considering a stand comprised mainly of Norway spruce with Silver fir (*Abies alba* Mill.) saplings in smaller-sized regeneration cohorts. Based on an height assessment of 327 fir saplings, which also included height increments of the last 5 years prior to the inventory, they concluded that the growth of these non-leading cohorts, each of them covering roughly 5% of a 1 *ha*-sized stand, can be described with the third yield class within a yield table set proposed by Hausser [2018]. Leading saplings at the same location, however, showed growth trends relating to the first yield class of the same framework instead. According to Hausser [2018], this difference in growth performance amounts to an average growth suppression (concerning the annual gross volume growth) of approximately 50%. It is important to point out that this estimation is based on fir and not spruce saplings, however, we would argue that growth characteristics of these two species, despite notable differences especially relating to their reaction capability in higher ages [Utschig, 2004], are close enough to tolerate this simplification.

In order to include this observation in our model, we compiled a simple logistic function (Equation 1), resulting in a growth reduction function sensitive to the age difference between the concerned cohorts and the gap size (Figure 2), which we applied in order to adjust the volume growth of non-leading cohorts (see main article, Section 2.2.2). It is worth noting that as soon a non-leading

cohort becomes dominant, e.g., due to the former leading cohort being harvested or affected by a disturbance, the volume growth of the remaining cohort is reset to its unsuppressed, yield table-based [Albert et al., 2022, Nuske et al., 2022] default.

$$r_{growth} = \frac{K}{1 + e^{r(\Delta age^{w_a} size_g^{w_g}) - s}} \tag{1}$$

where:

r_{growth}	is the volume growth reduction factor, relative to unsuppressed growth,
K	set to 1.049, is a shape parameter controlling the upper bound,
r	set to 0.06, is a shape parameter controlling the slope,
Δage	is the age difference between the leading and the suppressed cohort,
s	set to 50, is a shape parameter,
$size_g$	is the relative share of the suppressed cohort and
w_a and w_g	set to 1.1 and 2.5, are weights for Δage and $size_g$.

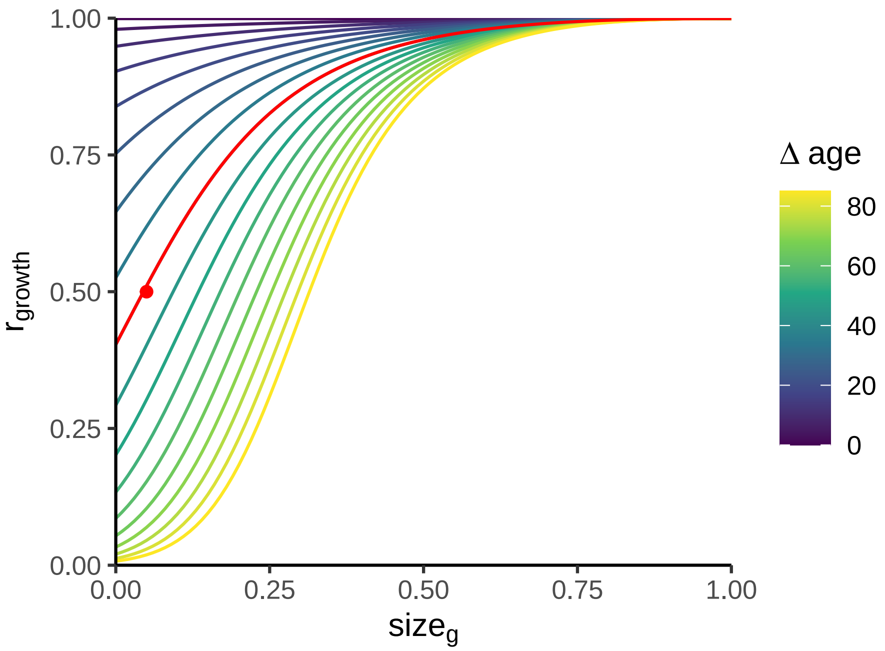

Figure 2: Volume-growth reduction function used during growth modeling (see main article, Section 2.2.2), compiled based on Knoke and Plusczyk [2001] and Hausser [2018]. The colors refer to the age difference between the concerned subdominant cohort and its dominant surrounding, whereas the x-axis refers to the size of the concerned subdominant cohort. The red line and dot indicate the observation derived from Knoke and Plusczyk [2001], indicating a sufficient alignment.

C: Details on the derivation of the growth acceleration factors applied in remaining leading cohorts

To adjust for the temporary growth acceleration occurring in the remaining leading cohorts after a gap cut or, after the final harvest or drop-out of a formerly leading cohort, we deployed a temporary, positive adjustment to their current volume growth (see main article, Section 2.2.2). Relations between stand density and relative growth are well studied in forest science (see for example Vilà et al. [2013], Forrester [2019], Baker [1953]), as they are important for adjusting

growth and yield predictions in forest management planning [von Gadow, 2005]. Many yield table frameworks, including the one used in this approach [Albert et al., 2022, Nuske et al., 2022] are thus equipped with correction factors allowing for an approximate adjustments for situations in which the density of a stand falls below (or exceeds) the tables default assumptions. In the case of Albert et al. [2022], the adjusted volume growth adj_{growth} is given as relative value in relation to the stocking density d_{stock} (Figure 3 a). It is worth noting that this relationship is not linear but concave, resulting in the volume growth being reduced slower than stock density once the latter is lowered, for instance, in our case during a gap cut activity. We can use this information to create a gap-size dependent volume growth acceleration function as follows:

First, if we assume the gap cut to cover a relative share of the total stand of size s_{gap}, the resulting stand density d_{stock} of the remaining stand after such a gap cut can be expressed as $1 - s_{gap}$. We can now compute the degree to which the stock density-dependent growth response given by Albert et al. [2022] (Figure 3 a) exceeds a strict linear relation between density and growth as $\frac{adj_{growth}}{d_{stock}}$, in which we can replace d_{stock} with the above $1 - s_{gap}$, resulting in Equation2:

$$acc_{growth} = \frac{adj_{growth}}{1 - s_{gap}} \tag{2}$$

where:

acc_{growth} is the volume growth acceleration factor, referring to a specific

s_{gap} which is the size of the gap, expressed as a share of the total stand.

By applying Equation 2 to all growth correction data points given by Albert et al. [2022], we receive a set of growth-acceleration estimates (Figure 3 b). After fitting a quadratic model to these values, we receive Equation 3, which we use as acceleration function for the volume growth in the remaining compartments.

$$acc_{growth} = -0.199s_{gap}^2 + 0.5261s_{gap} + 0.9975 \tag{3}$$

According to Equation 3, a gap cut of size $s_{gap} = 0.25$, i.e. covering 25% of the total stand, will result in a growth acceleration of 1.12 for the remaining cohort, meaning its volume growth would be adjusted to 112% of the yield table default.[1]

As the remaining trees utilize the new growing space quickly, the growth acceleration itself has to be temporary. We, thus, reset the volume growth back to the yield table default after 2 periods (10 years) after activation.

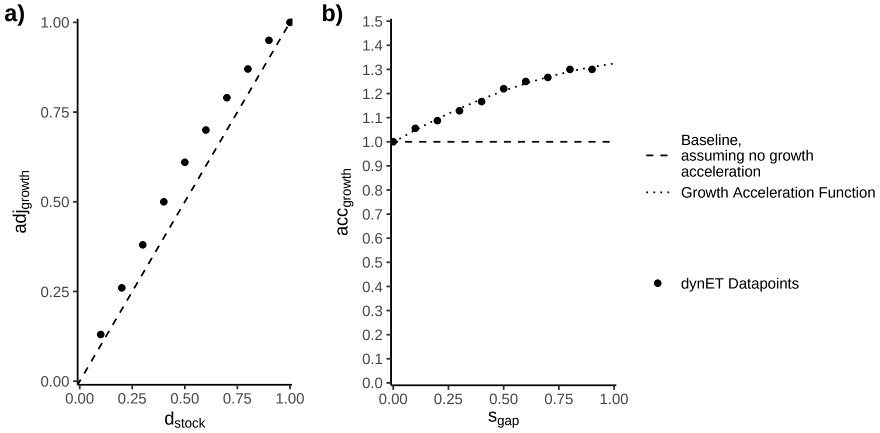

Figure 3: Source (a) and final shape (b) of the growth acceleration function used during growth modeling (see also main article, Section 2.2.2). Plot a) illustrates the original stock-density (d_{stock}) dependent correction factors for volume growth (adj_{growth}) as given by Albert et al. [2022]. By subtracting d_{stock} from 1, the equivalent gap size $size_g$ can be calculated (Plot b), and the overall acceleration in relative volume growth, referred to as acc_{growth}, can be calculated by dividing adj_{growth} given in Plot a) by their corresponding d_{stock}. By fitting a simple quadratic function to the resulting data points in b), we obtain a suitable growth acceleration function for the remaining cohorts (dotted line, see also Equation 3).

[1] Obviously acc_{growth} does not yet factor in the reduced area of the remaining compartment. As we explain in Section 2.2.2 (main article), this adjustment is carried out in a separate step.

D: Details on disturbance-type 4

In the main article, we classify disturbances occurring under the gap cut regimes into 4 types, based on the stand configuration at the moment of disturbance and its resulting state immediately after (see main article, Section 2.4). As the nature of type 4 disturbances might not be intuitively understood, a closer look at the chain of events necessary to create such an disturbance as well as the implications seems justified.

Disturbances of type 4 depend on a rather distinct series of events – they refer to the special case of matured former gap-cut cohorts, which no longer form the non-leading cohort in an uneven-aged stand. This case can occur a) once the leading cohort, following a prior gap cut, is harvested at the regular rotation age, and a later disturbance happens to only affect the former, now leading regeneration cohort, or b) once the same series of events is not triggered by regular harvest, but rather by a prior disturbance of type 3 taking out the leading cohort.

Due to the fact that disturbances of type 4 require such a distinctive (and thus less likely) series of events, they only represent a small fraction of the overall disturbance regime (see main article, Figure 5). However, *if* they happen, they tend to recover much faster than their counterparts under the clearcut baseline, mainly due to the limited area affected, and, thus, still exert a small, yet notable, effect on the average recovery time gains.

E: Remaining financial as well as carbon-oriented recovery-time gain distributions for all investigated regimes

In the main article, we presented two exemplary distributions of the recovery-time differences (δt_{rec}) of two gap-cut regimes (see main article, Section 3.1, Figure 5). We use this Supplement to present the remaining ones as well, both for the financial as well as the carbon sequestration dimension.

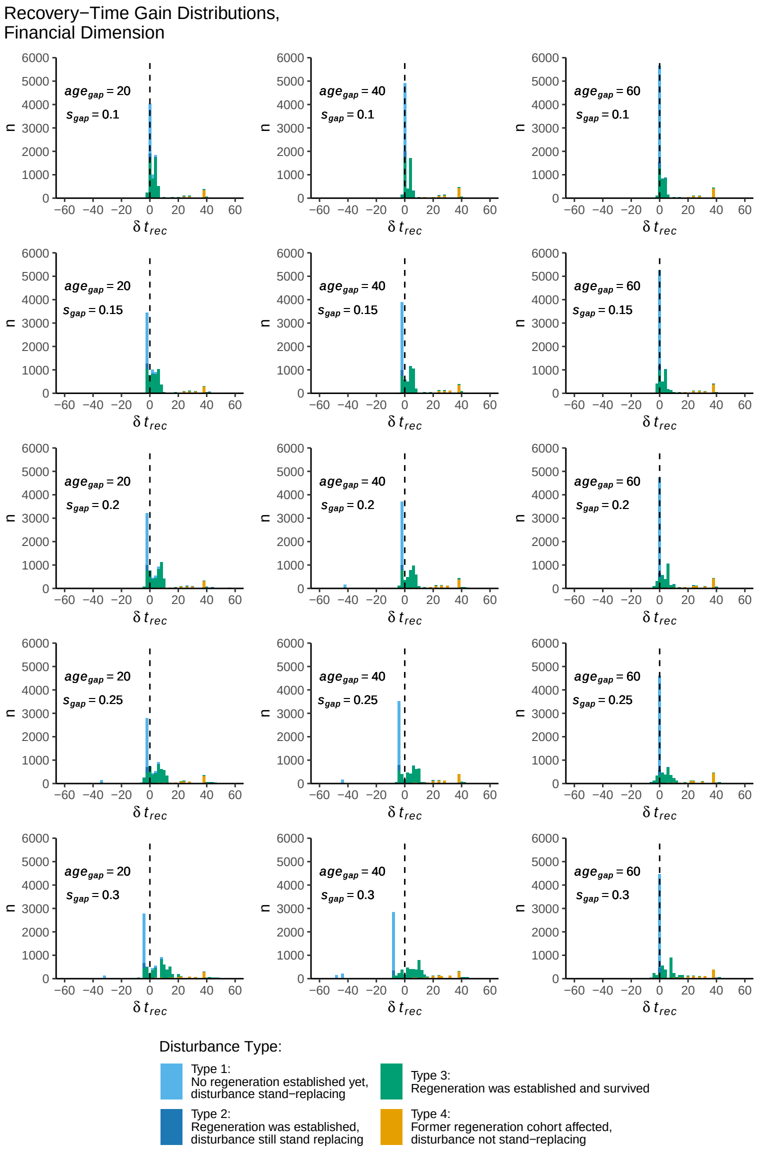

Figure 4: Distributions of recovery-time differences for all 15 gap-cut regimes relative to the clearcut baseline, concerning the financial dimension. For more details, see Figure 5 in main article.

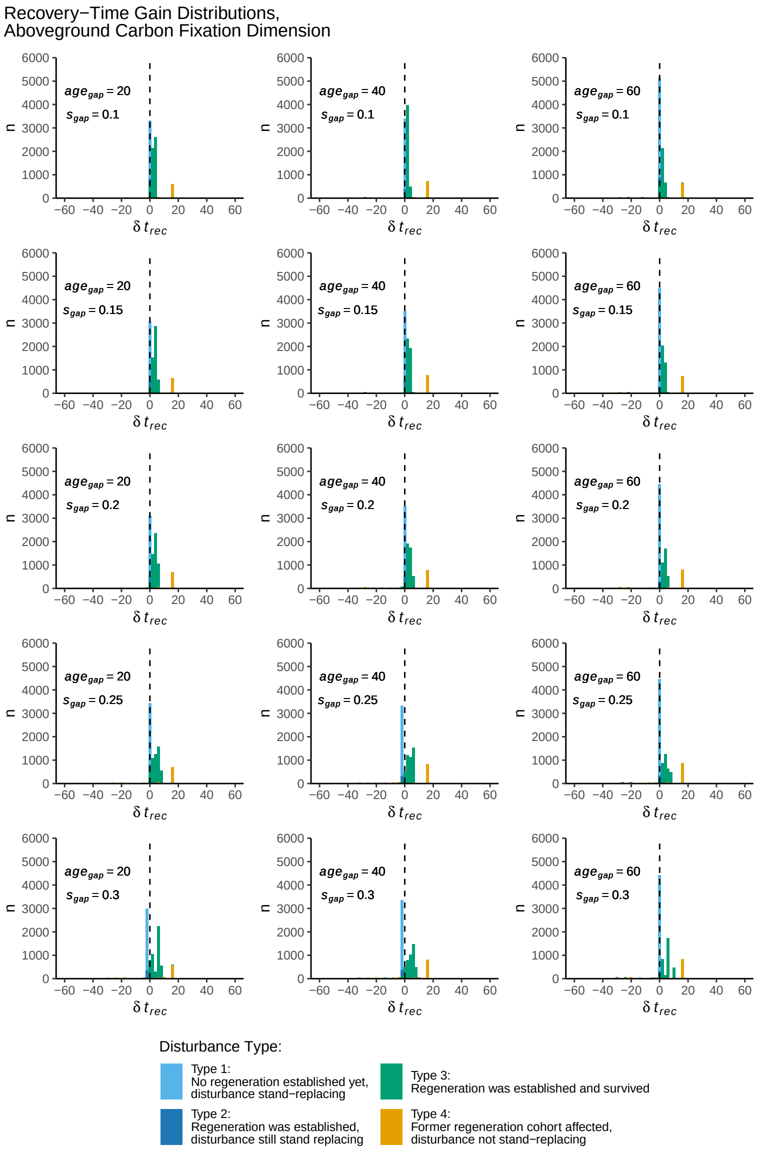

Figure 5: Distributions of recovery-time differences for all 15 gap-cut regimes relative to the clearcut baseline, concerning the aboveground carbon sequestration dimension. For more details, see Figure 5 in main article.

References

Matthias Albert, Jürgen Nagel, Matthias Schmidt, Ralf-Volker Nagel, and Hermann Spellmann. Eine neue Generation von Ertragstafeln für Eiche, Buche, Fichte, Douglasie und Kiefer. *Zenodo*, July 2022. doi: 10.5281/ZENODO.6827728.

F. S. Baker. Stand density and growth. *Journal of Forestry*, 51(2):94, 1953.

David I. Forrester. Linking forest growth with stand structure: Tree size inequality, tree growth or resource partitioning and the asymmetry of competition. *Forest Ecology and Management*, 447:139–157, September 2019. ISSN 03781127. doi: 10.1016/j.foreco.2019.05.053.

K. Hausser. Tannen-Ertragstafel. In *Hilfstafeln Für Die Forsteinrichtung*. Bayerisches Staatsministerium für Ernährung, Landwirtschaft und Forsten, 2018.

Thomas Knoke and Niels Plusczyk. On economic consequences of transformation of a spruce (Picea abies (L.) Karst.) dominated stand from regular into irregular age structure. *Forest Ecology and Management*, 151(1-3):163–179, October 2001. ISSN 03781127. doi: 10.1016/S0378-1127(00)00706-4.

Robert Nuske, Kai Staupendahl, and Matthias Albert. Et.nwfva: Forest Yield Tables for Northwest Germany and their Application. *zenodo*, November 2022. doi: 10.5281/ZENODO.7346918.

Hans Utschig. Wachstumskundliche Charakterisierung der Tanne. In *Beiträge zur Tanne*, number 45 in LWF Wissen, page 100. Bayerische Landesanstalt für Wald und Forstwirtschaft, Freising, 1 edition, 2004.

Montserrat Vilà, Amparo Carrillo-Gavilán, Jordi Vayreda, Harald Bugmann, Jonas Fridman, Wojciech Grodzki, Josephine Haase, Georges Kunstler, Mart-Jan Schelhaas, and Antoni Trasobares. Disentangling Biodiversity and Climatic Determinants of Wood Production. *PLoS ONE*, 8(2), February 2013. ISSN 1932-6203. doi: 10.1371/journal.pone.0053530.

Klaus von Gadow. *Forsteinrichtung: Analyse und Entwurf der Waldentwicklung*. Universitätsdrucke. Univ.-Verl. Göttingen, Göttingen, 2005. ISBN 978-3-938616-28-4.
